# Cardiac Orienting to Auditory Stimulation in the Fetus

**DOI:** 10.1177/2377960819861486

**Published:** 2019-07-24

**Authors:** Charlene Krueger, Cynthia Garvan

**Affiliations:** 1 University of Florida, Gainesville, FL, USA

**Keywords:** attention, auditory, developmental, fetus, orienting

## Abstract

The objective of this study was to longitudinally evaluate the cardiac response to auditory stimulation in fetuses born during their 28th gestational week. A longitudinal, within-subjects design allowed for interpretations of the cardiac response tracked from 28 to 38 weeks gestational age (GA). All mothers recited a short passage from 28 to 34 weeks GA, and their fetuses were tested at 28, 32, 33, and 34 weeks GA. Following discontinuation of maternal recitation at 34 weeks GA, testing continued at 36 and 38 weeks GA. Experimental subjects were tested with a recording of a female stranger speaking the assigned passage and control subjects tested with a novel passage. The cardiac response was evaluated visually and statistically based on the magnitude and duration of the changes in heart rate. Visually, the cardiac response transitioned from a minimal magnitude (<5 beats per minute) with short duration (<5 seconds) cardiac deceleration in both experimental and control subjects during testing from 28 to 38 weeks GA and was confirmed statistically. For all experimental subjects, however, a long duration or sustained (>5 seconds) cardiac deceleration of greater magnitude (>5 beats per minute) was detected during 34-, 36-, or 38-week test session and was confirmed using a computational algorithm in SAS. Further investigation into additional forms of auditory stimulation at different developmental time periods is needed.

## Introduction

At present, the vast majority of early developmental studies have focused on the subject's response to varied sources of sensory stimulation. As a potential window into early fetal learning capabilities and clinical well-being, this approach has resulted in an ever-enlarging mass of studies. While findings from these studies provide a basis for debates related to clinical well-being (Banta & Thacker, 2001) and how early evidence of learning comes about (Emory & Toomey, 1998), conclusions drawn by the researchers, in particular relating to attention and learning capabilities, have become confusing due to the ever-growing number of interpretations of the findings. The objective of this study was to longitudinally evaluate the cardiac response to auditory stimulation in fetuses born during their 28th gestational week. Using a longitudinal, within-subjects design, the fetal cardiac response to speech was tracked from 28 to 38 weeks GA and evaluated using both visual inspection and a computational review of the cardiac response.

### Fetal Response to Sound

The fetal response to sound is influenced by multiple factors. Of these, the age of the fetus and acoustical characteristics of the sound are fundamental. It is not until 28 weeks gestation that fetuses respond to changes in the frequencies of sound, three weeks after the cochlea and peripheral sensory end organs are structurally complete (Holst et al., 2005; Lasky & Williams, 2005). When the fetus is between 32 and 34 weeks GA, the cardiac response to white noise presented at 85 dB is sometimes an acceleration and sometimes a deceleration, whereas when the fetus is between 35 and 37 weeks GA, the cardiac response is consistently a deceleration (Morokuma et al., 2008; see also DiPetro et al., 1996; Gagnon, 1989; Groome, Mooney, Holland & Bentz, 1997). Human sleep-wake states or activity cycles begin developing before birth and significantly affect stimulus-elicited cardiac activity. Alternations between active and inactive states are not obvious before 30 weeks GA and periods of low heart-rate variability (LHRV) of < 5 beats per minute (bpm) are infrequent. After 32 weeks, GA activity/inactivity cycles become more discernible and around 36 weeks GA clearly differentiated sleep-wake states become apparent, 1F/sleep and 3F/awake are periods of LHRV, and 2F/sleep and 4F/awake are periods of high heart-rate variability (HRV; Nijhuis, Prechtl, Martin, & Bots, 1982). The term fetus, while in 1F/sleep and 3F/awake periods of LHRV, responds to high-intensity sound (>85 dB) with a cardiac acceleration which transitions to a cardiac deceleration as sound intensity decreases to a lower level (<85 dB; Lecanuet et al., 1986). This transition to a predominant cardiac deceleration, however, only occurs when the sound is within the frequency range of human speech. Higher frequencies result in a predominant cardiac acceleration (Lecanuet, Granier-Deferre & Busnel, 1988), which suggests that fetuses are differentially sensitive to frequencies in the speech range (see also Granier-Deferre, Ribeiro, Jacquet, & Bassereau, 2011b; Lecanuet, Granier-Deferre, Cohen, Le Houezec, & Busnel,1986; Lecanuet, Granier-Deferre, Jacquet, & Busnel, 1992).

When the variables noted earlier have been controlled, it has been possible to demonstrate that prior experience with the stimulus can also affect the cardiac response it elicits, that is, that late-term fetuses can learn about and remember specific sounds. For example, fetuses in the late prenatal period were consistently exposed to a descending pattern of music that was within the frequency range of speech, until they were 38 weeks GA. When these same subjects were 1-month old infants, they and a group of naive 1-month olds were tested with the descending pattern and with a different, ascending pattern. The descending pattern elicited a profound cardiac deceleration in infants that had been exposed to it before birth compared with a much lesser deceleration in naive infants. Infants that had been exposed to the descending pattern and naive infants, responded to the ascending pattern with the lesser deceleration (Granier-Deferre et al., 2011a). Thus, stimulus-elicited cardiac responses have been able to show that fetal exposure to a specific sound within the frequency range of speech late in gestation can have a powerful impact on auditory perception as long as 1-month after birth.

### Cardiac Orienting in the Fetus

When the fetal GA varies within and between groups and the history of exposure to maternal speech is absent or very brief (2 minutes), the predominant response to recorded maternal speech is a cardiac acceleration and the response to a stranger's recording of the same passage is a cardiac deceleration (Kisilevsky et al., 2003, 2009, 2011a; Kisilevsky & Hains, 2011b; Lee et al., 2007; Smith et al., 2007). In contrast, when the term fetus is provided with a longitudinal experience of hearing their mother speak a passage, the predominant cardiac response to playback of the passage is a cardiac deceleration. Furthermore, given the same longitudinal experience, when a novel speech passage is played back to the term fetus, the cardiac deceleration does not occur (DeCasper, Lecanuet, Maugeais, Granier-Deferre, & Busnel, 1994) and the same may hold true for the earlier fetus (Krueger, Holdtich-Davis, Quint, & DeCasper, 2004).

The conventional interpretation of this stimulus-elicited cardiac deceleration is that it represents the cardiac component of the orienting response (Clarkson, & Berg, 1983; Graham & Clifton, 1966; Kisilevsky, Fearon, & Muir, 1998; Porges, 1995, 2007; Reynolds & Richards, 2008; Schneirla, 1961). J. Richards (2001, 2015) detailed phases in the emergence of the cardiac-orienting response (COR) as the organism directs attention toward a *visual* stimulus based on earlier work by Graham and Clifton (1966), Lacey (1967), and Porges (1980, 1995, 2007).

J. Richards (2001) has described phases of attention, each with specific changes in heart rate as newborns and preterm infants respond to changes in *visual* stimulation. Changes in the cardiac response were hypothesized to vary with increasing age and to occur as the infant develops the ability to sustain attention. The first cardiac phase, *automatic interrupt*, is the infant's initial, reflexive response (decrease in heart rate) to transient changes in the environment. This phase may proceed to the second phase or terminate with a reflexive, heart rate acceleration. The second phase is *stimulus orienting*, during which a cardiac deceleration lasting 5 seconds is seen, indicates the likelihood of attention (rather than merely a reflexive response). This phase could also be referred to as emerging cardiac orienting. The third phase, *sustained attention*, is postulated to indicate cognitive processing that is clearly above the reflexive level. During this phase, HRV and movement are also diminished along with a greater magnitude and duration of cardiac deceleration. The fourth phase, *attention termination*, signals the return of heart rate and HRV to what they were prior to the first phase of visual attention. It is hypothesized that no further central nervous system processing occurs during this phase. At present, these four phases of the cardiac response to visual stimulation have been described in the newborn and preterm infant (Fox & Gellers, 1984; Frick & Richards, 2001; J. Richards, 1985, 1989a, 1989b) and are applied here in the fetus in response to auditory stimulation. Our goal is thus to provide a basic description of the cardiac response to speech that loans itself to an interpretation using this well-established theoretical approach related to when and how early attention processing/learning comes about.

## Materials and Methods

### Subjects

This study was approved by the university internal review board (IRB protocol #271-2007). Using a longitudinal, single subject design, eight fetuses were selected from a larger study in which 39 pregnant women and their fetus were tracked from 28 weeks GA to 38 weeks GA (Krueger & Garvan, 2014). Because this study is a longitudinal description of the emergence of the COR, only those individual fetuses whose mother attended all of the weekly testing sessions were included (see Table 1).

**Table 1.** Fetal Subjects.

Subject	Gender	Birthweight in grams	Delivery type	Apgar	
				1 minute	5 minutes
Experimental group					
10	Female	3,696	Vaginal	7	9
16	Female	3,570	C-Section	8	9
22	Female	3,481	C-Section	7	8
30	Male	3,225	Vaginal	7	8
Control group					
2	Female	3,316	Vaginal	9	9
6	Female	3,394	Vaginal	9	9
21	Female	3,417	C-Section	3	8
24	Female	3,598	C-Section	7	9

The target fetal age for admission into the study was 28 weeks GA, which was initially determined from the maternal last menstrual period and confirmed with either a first- or second-trimester ultrasound. Inclusion criteria consisted of (a) mothers 18 to 39 years of age (for legal participation in research and to avoid complications related to advanced maternal age, respectively), (b) mothers having undergone a first- or second-trimester fetal ultrasound to confirm GA, (c) English as the maternal native language (to maintain consistent stimulus parameters between the maternal voice and the recorded rhyme), and (4) maternal primipara (bearing a first child), ensuring that mothers did not have additional parenting responsibilities during the study.

Women and their fetus were excluded from the study if they were at increased risk for maternal, fetal, or infant morbidity/mortality as indicated by a history of endocrine, pulmonary, cardiovascular, or neurological problems. Specific medical conditions that excluded a potential participant included advanced maternal age, intrauterine growth retardation, oligo or polyhydramnios, maternal diabetes, maternal hypertension, and multiple gestations.

### Materials

#### Auditory stimulus

All mothers began participation in the study by speaking out loud one of the two different randomly assigned passages or nursery rhymes (Rhyme A and Rhyme B). Each rhyme took approximately 15 seconds to recite and was repeated 3 times in succession for 45 seconds (3 × 15 seconds = 45 seconds) twice a day from 28 to 34 weeks GA.

For longitudinal testing, a recording of each rhyme (Rhyme A or Rhyme B) was used. The recordings were done using a female stranger speaking the rhyme and evaluated for characteristics of *motherese* prior to use. Evaluation of the recordings passed the subjective test of reflecting slow speech with a wide variation of pitch. Objective measures of variation of pitch include the root mean square (RMS) of the auditory signals indicated that Rhyme A (minimum RMS power = −93.76 dB; maximum RMS power = −9.81 RMS; average RMS power = −29.21 dB; total RMS power = −24.99 dB) and Rhyme B (minimum RMS power = −93.51 dB; maximum RMS power = −7.41 RMS; average RMS power = −23.87 dB; total RMS power = −20.21 dB) were not statistically different, according to the Mann–Whitney *U* test.

At each test session, recordings were played back to the fetus over a 2.5-in. speaker positioned approximately 20 cm above the mother's abdomen. Overall, stimulus intensity at the abdomen was 76 dB (±5 dB), as measured by a Brüel & Kjær sound-level meter (model #2239) using the Leq of A-weighted sound pressure levels (Gray & Philbin, 2000). This sound level was chosen because it produces an intrauterine sound level of approximately 56 dB (Gerhardt, 1989; D. Richards et al., 1992) and is consistent with the sound level used in prior studies with similar methodology (Krueger, Cave, & Garvan, 2014; Krueger & Garvan, 2014; Krueger et al., 2004).

#### Data collection system

Fetal heart rate, HRV and movement were obtained using a cardiotocograph (Corometric model 170) fetal monitor. The analog signal from the fetal monitor output was recorded in two different ways: a paper monitor strip and digitized for conversion to text format. The paper monitor strip was produced immediately from the fetal monitor at a speed of 3 cm/minute using a fetal Doppler ultrasound method (4 Hz) to detect heart periods and movement in the fetus. Heart periods were also sampled from the analog port at a sampling rate of 500 Hz and digitized for conversion to text format using Matlab software (The MathWorks, release 14). The files were then transferred from laptop (Dell Inspiron 8100) to a PC (Dell Optiplex GX270) for subsequent graphic display and statistical analyses. Only those files in which >95% of the signal was detected were included in the final analyses.

### Procedure

As detailed in the larger study (Krueger & Garvan, 2014) to test for the emergence of cardiac orienting, fetuses were tested at 28, 32, 33, and 34 weeks. At 28 weeks GA, neither the experimental nor control fetuses had prior exposure to the passage of speech before testing. To test for the retention of cardiac orienting, maternal recitation was discontinued at 34 weeks and testing occurred at 36 and 38 weeks GA. Experimental group fetuses were tested with a recording of the passage assigned to their mothers for recitation (either Rhyme A or Rhyme B), while fetuses control group fetuses were tested with a passage their mothers had not been reciting (either Rhyme A or Rhyme B).

#### Maternal recitation

Before mothers began their recitation at 28 weeks GA, a research assistant demonstrated how to speak using motherese or using slower speech with a wider variation of pitch. Although the effect of motherese on the fetus is unknown, newborns have been shown to systematically prefer human speech with these characteristics (Cooper & Aslin, 1990). All mothers were asked to choose two convenient times—one in the morning and one in the afternoon—in which to recite the assigned rhyme. Some variations in recitation times were expected because of changes in family and work routines; thus, mothers completed a written home log and only those returning there logs were included in the analyses.

Variables controlled for during maternal recitation were as follows: (a) time since last meal, (b) maternal position during recitation, (c) additional sensory stimulation during recitation, and (d) the fetal sleep-wake state. During recitation, mothers were asked to eat at least 15 minutes prior to testing, sit in a semirecumbent, left lateral tilt position, and not rub or touch their abdomens. These procedures controlled for increases in fetal body and breathing movements following maternal eating and avoided the possibility of decreased blood flow to the fetus if the mother lies flat on her back and allowed for additional sensory stimulation when the recitation was performed.

While a consistent fetal state or sleep-wake period during recitation cannot be verified without fetal monitoring, mothers were asked to begin recitation only after they had felt no fetal movement for at least 1 minute. It has been found that mothers detect approximately 90% of all fetal movements as compared with ultrasound (Conners, Natale, & Nasello-Paterson, 1988). One minute was chosen in order to maintain consistency with the sleep-wake criteria for the test sessions.

#### Test sessions

Testing was conducted in the General Clinical Research Center located within Shands Teaching Hospital (now referred to as UF Health at Shands) and was begun at least 15 minutes after the mother had eaten; thus, mothers were asked to eat prior to arriving. As with maternal recitation described earlier, mothers were placed in a semirecumbent, left lateral position to avoid supine hypotension and mothers were requested not to touch their abdomen during testing. For the mother's comfort, data were collected for a maximum of 30 minutes.

Recordings were broadcast over a 2.5-in. speaker positioned 20 cm above the mother's abdomen and were not initiated until the fetus was determined to be in a quiet period of activity using quiet-activity criteria established by Arduini, Rizzo, and Romanini (1995). According to these criteria, the fetus is in a quiet period when (a) fetal HRV is diminished (<10 bpm), (b) no fetal movement is detected, and (c) no heart rate accelerations of 15 bpm are noted. Due to the immaturity of the central nervous system, fetuses <30 weeks' GA spend less time with periods of HRV of <5 bpm than fetuses near term age. Moreover, fetal behavioral sleep-wake states (1F, 2F, 3F, and 4F) and their associated patterns of HRV are not apparent until 36 weeks GA (Nijhuis et al., 1982). Thus, across-age comparisons of stimulus-induced heart rate changes could be made. For the mother's comfort, we required that these criteria be met within 30 minutes of initiation of fetal monitoring. If the criteria were not met, no stimuli were presented, and the session was terminated. Due to the potentially small magnitude of the cardiac response and the need to maintain consistent quiet period criteria, test session results were not analyzed if the standard deviation of fetal heart rate was ≥10 bpm or if the fetus moved during the 15-second prestimulus period.

To control for maternal reactions during test sessions, a number of procedures were followed: (a) mothers were asked not to speak or touch their abdomens, while the measurements were being taken and the rhymes were played or spoken; (b) mothers listened through headphones to a predetermined musical set, while the recorded rhymes were played to the fetus (so mothers could not inadvertently hear the rhyme, which could produce an unrelated maternal response); (c) the monitor was positioned so mothers could not see the heart rate tracing (van den Bergh, 1992); and (d) the transducers for detecting fetal heart beat and movement were gently placed so fetuses were not disturbed.

### Data Analysis

Data collected during test sessions were obtained from a 15-second prestimulus baseline followed by a 15-second stimulus period when the passage or nursery rhyme was played back to the fetus.

#### Preliminary data analyses

For all observations, the individual prestimulus baseline was evaluated for confirmation of quiet-activity criteria using the standard deviation of the prestimulus heart rates and deemed stable if less than 10 bpm.

#### Transformation of heart rate using difference scores

Once stability of the baseline was met, the prestimulus and stimulus heart rates were transformed using difference scores, allowing for an evaluation of the cardiac response for each subject. Difference scores were created by using the 15-second prestimulus mean and subtracting this mean from each 1-second heart rate of the overall 15-second prestimulus and stimulus periods. The use of difference scores assists in detection of changes in heart rate by reducing variability in the baseline.

#### Visual review

Difference scores for each subject were then evaluated visually. Because the COR is detailed as a deceleration, subjects with an increase in the difference scores (cardiac acceleration) were categorized as no cardiac orienting. For subjects with a decrease in difference scores, the magnitude and duration of the cardiac deceleration were then defined based on criteria previously described by Richards' et al. for the first three phases of visual attention. The fourth phase, *attention termination*, signaled a return of heart rate to what it was prior to the first phase of visual attention so did not have the same dimensions (magnitude and duration) and therefore was not included in the review.

**Table:** 

		Magnitude	Duration
Phase 1	Automatic interrupt	<5 bpm	Not evaluated
Phase 2	Stimulus orienting	≥5 bpm	<5 seconds
Phase 3	Sustained attention	≥5 bpm	≥5 seconds

*Note*. Phase 4 or attention termination was not included in review. bpm = beats per minute.

#### Computational analysis review

The magnitude and duration described in “Visual review” section were then used to develop a computational algorithm implemented in SAS software (Cary, N.C.) that differentiated between each of the phases of attention. This review provided numerically based confirmation of what was examined visually.

## Results

Using a longitudinal data set, the individual fetal cardiac response to speech was evaluated both visually and statistically from 28 to 38 weeks GA.

### Visual Review

#### 28 weeks GA

**Figure 1.**
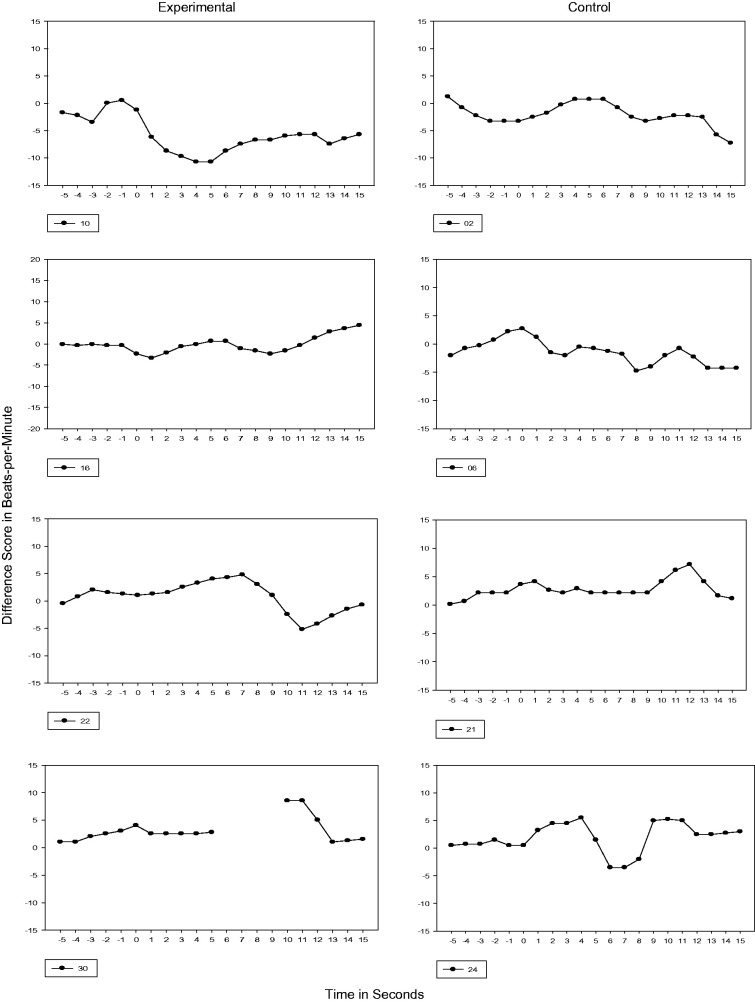
28 Weeks GA Cardiac Response. Vertical axis is heart rate transformed using difference scores. Each figure represents the 5s prestimulus baseline period and a 15s stimulus period.

At 28 weeks GA, neither the experimental nor control fetuses had prior exposure to the passage of speech before testing. During this first test session, Phase 1 could be seen in all experimental and control subjects except one control fetus (Subject 21) and 25% transitioned to Phase 2 or a ≥5 bpm cardiac deceleration with <5-second duration. For Subject 10, the duration of the cardiac deceleration was sustained for longer than 5 seconds (see Figure 1).

#### 32, 33, and 34 weeks GA

**Figure 2.**
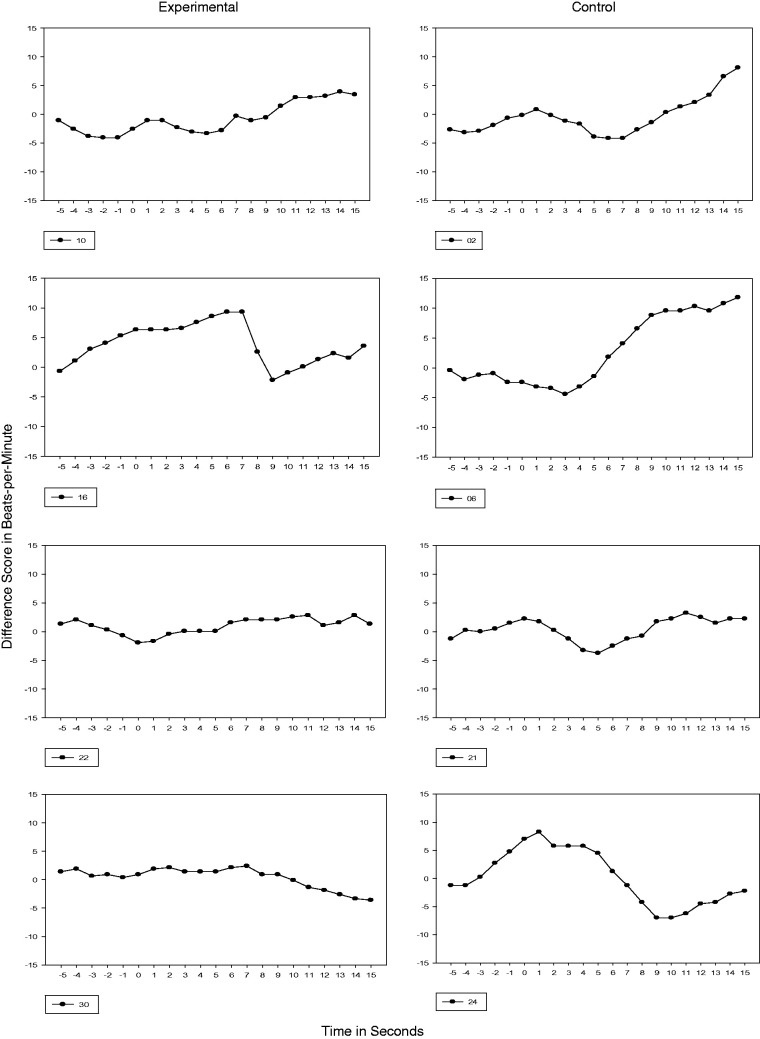
32 Weeks GA Cardiac Response. Vertical axis is heart rate transformed using difference scores. Each figure represents the 5s prestimulus baseline period and a 15s stimulus period.

**Figure 3.**
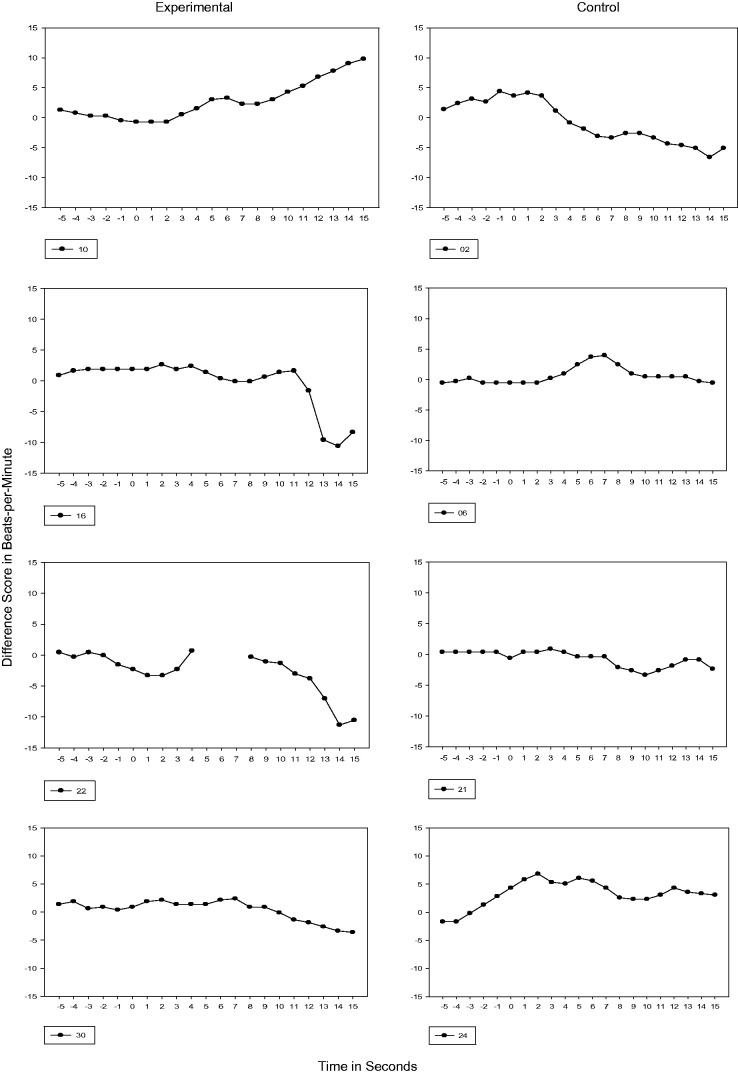
33 Weeks GA Cardiac Response. Vertical axis is heart rate transformed using difference scores. Each figure represents the 5s prestimulus baseline period and a 15s stimulus period.

**Figure 4.**
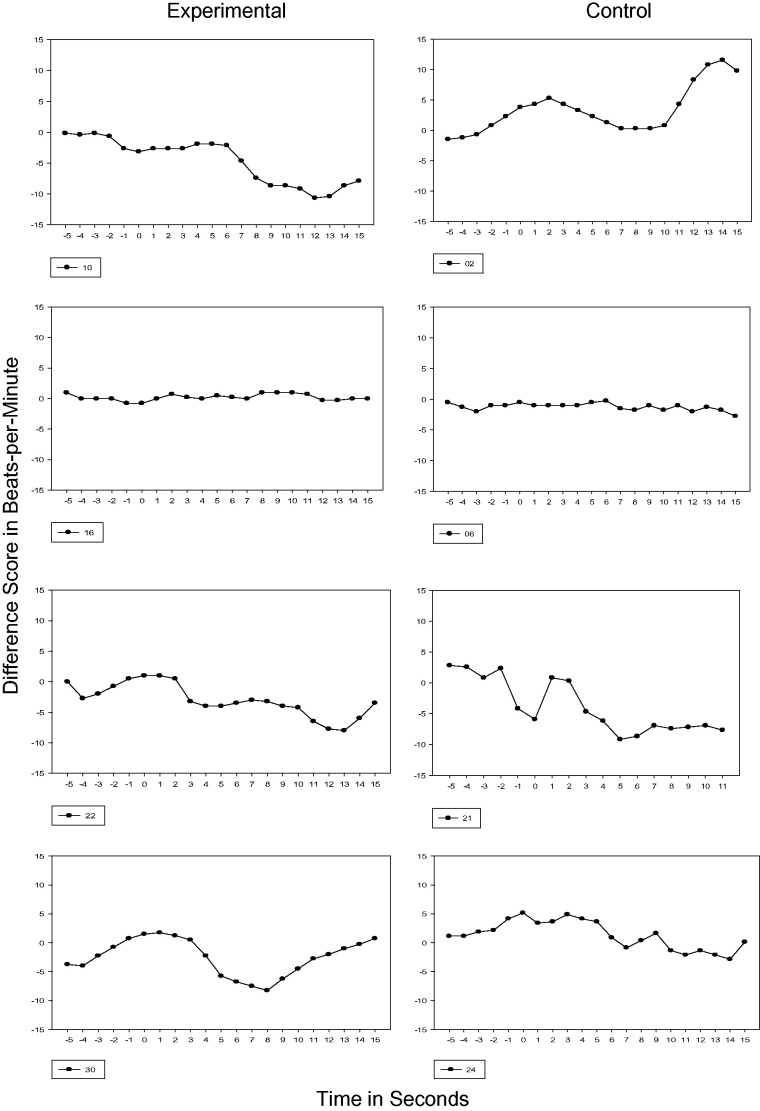
34 Weeks GA Cardiac Response. Vertical axis is heart rate transformed using difference scores. Each figure represents the 5s prestimulus baseline period and a 15s stimulus period.

Test sessions performed at 32, 33, and 34 weeks GA were completed following 4, 5, and 6 weeks of maternal recitation of the passage, respectively. By 33 and 34 weeks GA, all experimental fetuses demonstrated a transition to Phase 2 and 50% at 34 weeks GA demonstrated Phase 3 or a sustained cardiac deceleration. In contrast, one control subject never transitioned beyond Phase 1 (Subject 6) and 50% of the control subjects transitioned to Phase 2. For one control subject at 34 weeks GA (Subject 21), the cardiac response transitioned to sustained attention or Phase 3 (see Figures 2 to 4).

#### 36 and 38 weeks GA

**Figure 5.**
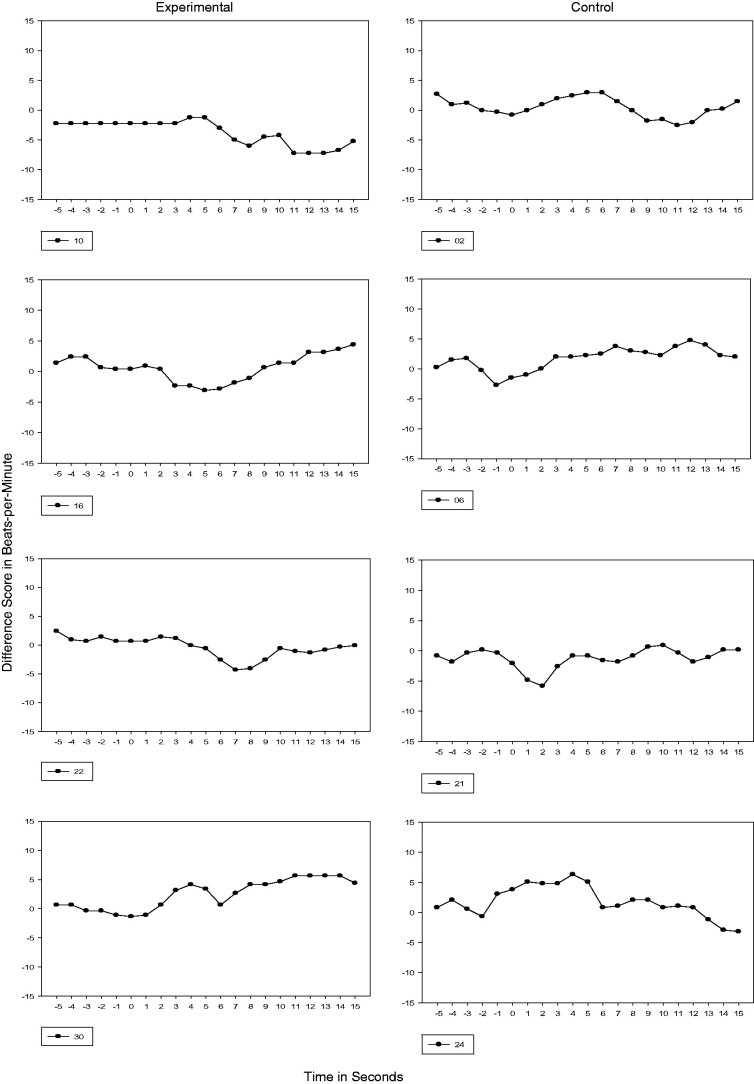
36 Weeks GA Cardiac Response. Vertical axis is heart rate transformed using difference scores. Each figure represents the 5s prestimulus baseline period and a 15s stimulus period.

**Figure 6.**
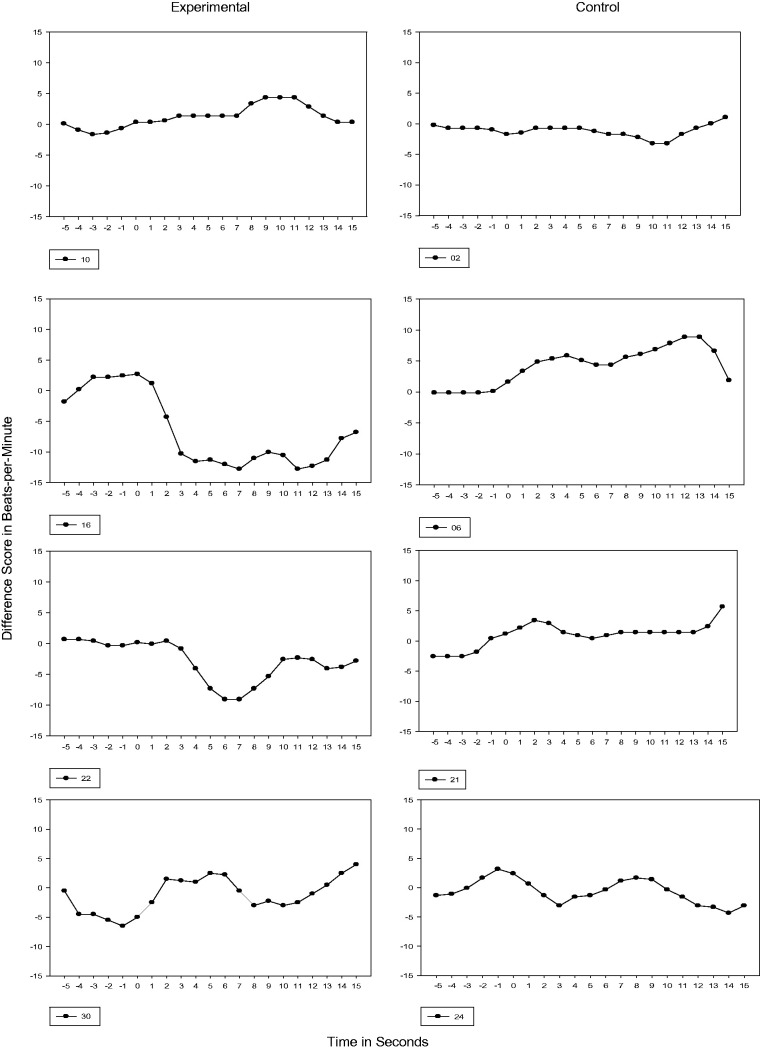
38 Weeks GA Cardiac Response. Vertical axis is heart rate transformed using difference scores. Each figure represents the 5s prestimulus baseline period and a 15s stimulus period.

Because mothers discontinued reciting the passage at 34 weeks GA, subsequent testing performed at 36 and 38 weeks GA were probes for retention of cardiac orienting. Of note is that by 38 weeks GA, all experimental subjects demonstrated a transition in their cardiac response from Phase 1 to Phase 3. The cardiac response for control subjects, however, was predominantly Phase 1 with one subject (Subject 21) transitioning to Phase 2 (see Figures 5 and 6).

### Computational Review

Using the same criteria applied during the visual review, each phase was defined and assigned a number (*no response* = 0; *Phase 1* = 1; *Phase 2* = 2; *Phase 3* = 3). A review based on the computer-developed algorithm confirmed all visual interpretations. A longitudinal summary of the phasic change in the cardiac response (confirmed both visually and computationally) is shown in Table 2.

**Table 2.** A Longitudinal Representation of Cardiac Orienting.

Subject	28	32	33	34	36	38
Experimental group						
10	3	1	1	3	3	0
16	1	1	2	1	1	3
22	2	1	2	2	1	3
30	0	1	1	3	1	2
Control group						
2	2	1	2	0	1	1
6	1	1	1	1	1	0
21	0	1	1	3	2	0
24	1	2	0	1	1	1

## Discussion

This study contributes evidence for the application of Richards et al.'s phases of visual attention to stimulation provided via sound in the fetus. A transition in heart rate from Phase 1 to Phase 2 was demonstrated in both experimental and control subjects from 28 to 38 weeks GA. Phase 3 or sustained attention was demonstrated in all experimental subjects between 34 and 38 weeks GA with one control subject demonstrating sustained attention at 34 weeks GA. All visual interpretations were supported using a computer algorithm implemented in SAS.

While (once definitions were decided upon) the visual interpretation was straight forward, the practice of reviewing each individual subject's tracing could be very time consuming and is subjective. With the addition of SAS programmed with the same criteria used for the visual interpretation, not only can this reduce the time necessary to review individual subject tracings, but numerical assignment to different phases of attention can be made allowing for a more efficient examination of interactions between other variables. For example, this method could allow for a more efficient examination of interactions between gender or ethnicity and the different phases of attention.

The data reported here are taken from a larger sample in which only grouped measures were examined (Krueger & Garvan, 2014). In this larger sample, a transition to Phase 3 or sustained attention was not shown until 38 weeks GA. What is therefore revealed here by conducting the single subject review of the data is the individual variability at which attention directed toward sound progresses. For example, while not shown in the grouped measures, a single subject review revealed that two subjects showed sustained attention as early as 34 weeks GA. Why these subjects would respond earlier than the others is currently not known; however, this study suggests that single subject reviews may be useful in increasing our understanding of additional factors involved in the emergence of attention.

This study provides further support for the ability of the early fetus to direct attention toward an auditory stimulus. In another study conducted by Krueger et al. (2001) with preterm fetuses in which mothers were asked to recite a nursery rhyme from 28 to 34 weeks' fetal gestation, it was found that a COR was detected by 34 weeks of age and, as in this study, no COR was detected prior to functional maturity of the fetal autonomic nervous system at approximately 32 weeks GA (Dalton, Phil, Dawes, & Patrick, 1983; Holmes, 1986). Taken together with the findings reported here, this suggests that maturation is an important factor to investigate in the future.

Limitations to the study include the subjective nature of visual reviews of the fetal monitor tracing, the need to balance groups by gender, and a method to characterize the prestimulus baseline are needed. The SAS computer algorithm assists in overcoming the subjective nature of single subject review. Balance between groups by gender can be overcome with a prospective design. The prestimulus baseline for one control subject displayed sustained attention; however, a descending trend in the prestimulus baseline was present. In the future, careful control of the baseline trend will be needed. In that, a method to determine whether the baseline is trending downward (thus, potentially increasing the likelihood of a cardiac deceleration with stimulus onset) will be needed.

Criteria given by Richards et al. for phases of visual attention were used in this study to establish the magnitude and duration of the cardiac deceleration for Phase 3 (sustained attention) at ≥5 bpm lasting ≥5 seconds duration. While criteria for the phases of visual attention were not as specific as those reported here, the specificity allowed for computer algorithm confirmation of the visual review of individual subject data and largely differentiated between the experimental and control group response to auditory stimulation. Given experience with the auditory stimulus, all experimental subjects showed a cardiac deceleration consistent with sustained attention; however, the cardiac deceleration emerged at different GAs. Is it that the phases defined here need further definition? In particular, how we differentiate between Phase 4 (attention termination) and no response (no change in cardiac response from baseline) and is it a useful description of the emergence of attention and learning capabilities have not been developed yet?

**Figure 7.**
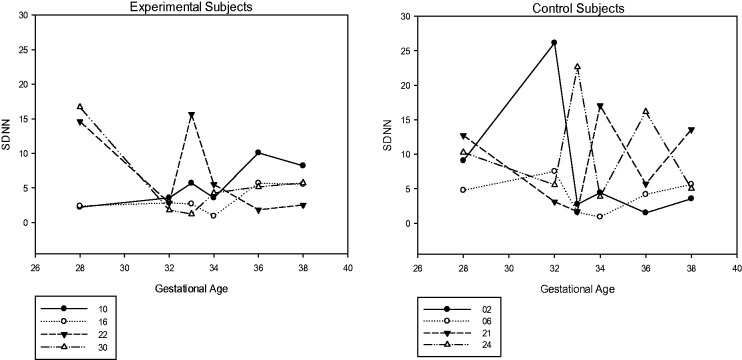
Heart Rate Variability During Stimulus Period Vertical axis is heart rate variability using the SDNN of heart periods during the 15s stimulus period.

Furthermore, Richards et al. (2001) report diminished HRV and movement during sustained attention. These variables were also considered here. A post hoc evaluation of HRV showed some compression in the overall range of HRV (see Figure 7); however, no apparent decrease in movement was shown (see Table 3). In the future, variations in how these variables (HRV and movement) are obtained and subsequently defined are needed. For example, a frequency-based analysis of heart periods taken from a transabdominal electrocardiogram signal may prove more useful than that employed here (time-based analysis) and differentiation between gross motor and fine motor movement may be needed.

**Table 3.** Fetal Movement Count During Stimulus Playback.

	28 weeks	32 weeks	33 weeks	34 weeks	36 weeks	38 weeks
Experimental subject						
10	0	1	2	1	4	3
16	1	3	0	0	1	1
22	6	2	5	4	0	0
30	4	2	0	6	6	4
Control subject						
2	1	2	0	2	1	1
6	0	2	0	0	1	1
21	6	1	1	4	1	5
24	1	0	7	2	2	1

In summary, the usefulness of a within-subject subject visual review combined with a computer algorithm implemented in SAS to confirm objectively what is seen, we believe, has been demonstrated here and holds promise for future research. Additional study, however, will be needed to differentiate between a stable (no change), ascending or descending baseline prior to stimulus onset, a frequency-based analysis of HRV free of signal averaging, balance by gender between groups, and closer evaluation of fetal movement (possibly under ultrasound). With further definition of these variables and repeated demonstrations of the individual cardiac response to stimuli, the method used here may prove useful in determining whether a larger dose or more frequent, longer repetitions of speech by mothers would have resulted in earlier detection of sustained cardiac orienting and potentially why some variations in the onset of sustained orienting occurs. It is cautiously concluded here, however, that this interpretation of the fetal cardiac response to speech has provided a basic longitudinal, within-subject description and holds prospect for the investigation of what impact additional forms of auditory stimulation have on the developing fetus.
